# Editorial: Single cell analysis in endocrinology, reproduction, and metabolism

**DOI:** 10.3389/fendo.2023.1282987

**Published:** 2023-10-23

**Authors:** Francisco Dominguez, Chellakkan S. Blesson

**Affiliations:** ^1^ IVIRMA Global Research Alliance, IVI Foundation, Instituto de Investigación Sanitaria La Fe (IIS La Fe), Valencia, Spain; ^2^ Department of Obstetrics and Gynecology, Baylor College of Medicine, Houston, TX, United States; ^3^ Family Fertility Center, Texas Children’s Hospital, Houston, TX, United States

**Keywords:** reproduction, metabolism, endocrinology, personalized medicine, single cell analysis

The field of endocrinology, reproduction, and metabolism has long been fascinated by the intricate interplay of cellular dynamics that underlie complex physiological processes. Exploring the complex cellular dynamics that govern these processes has been a pursuit of scientists and researchers for decades. Recent advancements in single-cell analysis techniques have propelled us into a new era of understanding, allowing us to dissect the intricate molecular underpinnings of these systems at unprecedented resolution. In this Editorial, we delve into the implications and potential of single-cell analysis in elucidating the complexities of these fields, focusing on the remarkable insights garnered from three seminal articles.

## Understanding placental dynamics in gestational diabetes mellitus

Gestational diabetes mellitus (GDM) presents a significant challenge to maternal and fetal health. Increasing evidence has suggested that the placenta, a central player in pregnancy, is intricately involved in GDM’s pathogenesis. However, unravelling the complexities of the human placenta has proven elusive until the advent of single-cell RNA sequencing (scRNA-seq). By examining cellular signatures and transcriptomes, Yang et al. have illuminated the diverse cell subtypes and their subtype-specific marker genes within the placenta. This comprehensive cell atlas not only enhances our understanding of the placenta’s cellular function but also offers insights into GDM’s molecular underpinnings. The implications of this research extend beyond diagnosis, potentially informing novel strategies for GDM treatment and prevention.

## Deciphering the cellular basis of erectile dysfunction

Erectile dysfunction (ED), a condition often rooted in the intricate connections between reproduction and metabolism, has remained a clinical challenge. The corpus cavernosum, a critical component of the male reproductive system, harbours crucial insights into ED. Employing single-cell RNA sequencing, Fang et al. have delved into the cellular tapestry of the corpus cavernosum in patients with severe ED. This meticulous analysis has unveiled a myriad of cell populations, revealing the complex cellular heterogeneity inherent to this tissue. Furthermore, this study has illuminated potential contributors to ED, including fibrosis and inflammation. By comprehensively unravelling the cellular landscape, this research not only advances our knowledge of ED’s cellular basis but also lays the foundation for individualized treatment strategies.

## Epigenetic regulation and prognostic implications in hepatocellular carcinoma

The intricate interplay between metabolism and malignancy is exemplified in hepatocellular carcinoma (HCC). Epigenetic regulation, particularly histone modifications, influences gene expression patterns and contributes to HCC’s development. Leveraging the power of single-cell RNA sequencing, Fan et al. have probed the role of histone phosphorylation in HCC prognosis. By constructing a prognostic risk signature based on the expression of specific genes, they have provided insights into the intricate interplay between histone modifications, the tumor microenvironment, and disease progression. This not only aids in prognostic assessment but also deepens our understanding of the molecular mechanisms underlying HCC’s complexity.

## Towards personalized medicine and tailored interventions

As these three articles collectively showcase, single-cell analysis is revolutionizing our understanding of the intersections between endocrinology, reproduction, and metabolism. The insights garnered from scrutinizing individual cells transcend traditional methodologies, uncovering nuances that were previously hidden. These studies collectively emphasize the significance of dissecting cellular heterogeneity, unravelling gene expression patterns, and deciphering the dynamic interactions between cells. Also, these studies underscore the value of dissecting cellular heterogeneity and interactions to glean novel insights into diseases that profoundly impact human health. Such a comprehensive approach holds immense promise in the realm of personalized medicine, offering tailored interventions for different conditions and diseases.

In the broader landscape of scientific progress, these endeavours represent more than individual studies. They are pieces of a larger puzzle that, when integrated, illuminate the complex web of cellular interactions underlying health and disease. As the field of single-cell analysis continues to evolve, the depth of knowledge gained will undoubtedly inform clinical approaches and therapeutic strategies. From unravelling the mechanisms of pregnancy complications to deciphering the cellular basis of sexual health and probing the epigenetic intricacies of cancer, single-cell analysis is a powerful tool that propels us toward a future of personalized, precision medicine.

In closing, the symbiotic relationship between science and technology continues to reshape the boundaries of human understanding. The uncovering of endocrinology, reproduction, and metabolism through the lens of single-cell has revolutionized our ability to dissect the intricacies of these fields, shedding light on previously uncharted territories and offering profound insights into various physiological and pathological phenomena. Single-cell technologies have enabled us to investigate the interactions and functions of a single cell in a way that was unthinkable a decade ago. Single-cell technologies are still in their infancy and require further breakthroughs to understand molecular and metabolic interactions. Various emerging technologies will pave ways to investigate intracellular signalling which may lead to novel discoveries.

## Author contributions

FD: Writing – original draft, Writing – review & editing. CB: Writing – original draft.

